# A Case of Fish Sausage Anaphylaxis Induced by Epicutaneous Sensitization to Carmine Contained in Eyeshadows: The Effect of Chelation on Carmine Allergy

**DOI:** 10.1155/2024/1057957

**Published:** 2024-09-11

**Authors:** Maiko Yamaura, Yuriko Iwahashi, Eri Hashimoto, Jun Miura, Yuri Murayama, Sachiko Koshikawa, Naoko Inomata

**Affiliations:** Department of Dermatology Showa University School of Medicine, Tokyo, Japan

## Abstract

Carmine is an aluminium and/or calcium-chelated complex form of carminic acid (CA), which is derived from the *Dactylopius coccus* extract (cochineal), and is globally used as a red-colourant in foods and cosmetics. Although several allergens in carmine allergies, such as CC38K, have been reported, it remains unknown whether chelation affects the allergenicity of carmine. We report a case of Japanese fish sausage (*Gyoniku Sausage*) anaphylaxis induced by epicutaneous sensitization to carmine contained in eyeshadows. In addition, we report on the effect of chelation on carmine allergy. A 32-year-old woman had experienced itching, wheals, and swelling of her eyelids immediately after applying pink eyeshadows, which contained carmine, on several occasions for 3 years. Two months ago, she developed itching, wheals, and swelling on her whole body, especially her eyelids, and dyspnea immediately after ingesting fried pink fish sausages, which contained cochineal. In skin prick tests (SPTs) with all ingredients ingested in the two episodes of anaphylaxis, only fish sausage was positive. SPT was also positive for carmine. In IgE-immunoblotting using the eyeshadow and fish sausage, the patient serum IgE was bound to three protein bands at approximately 86, 114, and 130 kDa. In addition, IgE-immunoblotting using the carmine showed a broad band at 86–130 kDa, which were consistent with those using the eyeshadow and fish sausage, whereas there is no band using CA. Interestingly, the protein bands using the eyeshadow and carmine were diminished by preincubation in the presence of ethylenediaminetetraacetic acid (EDTA) as a chelating agent. The results indicated that the causative allergens of carmine contained in the eyeshadows could be dechelated by EDTA, reducing its allergenicity. In conclusion, carmine contained in cosmetics can cause epicutaneous sensitisation and consequently can induce food anaphylaxis. To prevent sensitisation in carmine allergy, the effect of chelation on allergenicity of carmine should be considered.

## 1. Introduction

Carmine is an aluminium and/or calcium-chelated complex form of carminic acid (CA), which is derived from the *Dactylopius coccus* extract (cochineal), and is globally used as a red-colourant in foods and cosmetics. Although several allergens in carmine allergies, such as CC38K, have been reported, it remains unknown whether chelation affects the allergenicity of carmine [1–5]. We report a case of Japanese fish sausage (*Gyoniku Sausage*) anaphylaxis induced by epicutaneous sensitization to carmine contained in eyeshadows. In addition, we report on the effect of chelation on carmine allergy.

## 2. Case Report

**Figure 1:**
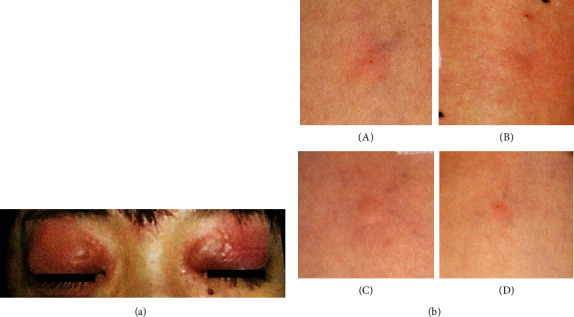
(a) Redness and swelling of eyelids induced after ingesting the fried fish sausages. (b) Results of skin prick tests with the eyeshadow, the fish sausages, carmine, and carminic acid. Skin prick tests with (A) the pink eyeshadow 1% in saline (mean diameter of wheal, 4.4 mm), (B) the fish sausages 10% in saline (7.4 mm), (C) the carmine 0.1% in phosphate buffered saline (PBS), (4.9 mm), and the carminic acid 0.1% in PBS, (3.6 mm) were positive. The mean diameter, which was induced by 10 mg/ml histamine hydrochloride as positive control, was 6.6 mm, whereas the mean diameter, which was induced by saline as negative control, was 1.1 mm. The elicited response was considered positive if the largest allergen-induced wheal diameter was ≥3 mm.

A 32-year-old woman had experienced itching, wheals, and swelling of her eyelids immediately after applying pink eyeshadows (Kana-labo, Tokyo, Japan), which contained carmine, on several occasions for 3 years. Two months ago, she developed itching, wheals, and swelling on her whole body, especially her eyelids, and dyspnea immediately after ingesting fried pink fish sausages (Maruha Nichiro, Tokyo, Japan), which contained cochineal (Figures 1(a)). She was administered with adrenaline i.m. and corticosteroid i.v. in an emergency room and was admitted for two days. In hospitalization, specific IgE measurements using ImmunoCAP (Thermo Fisher Scientific, Waltham, MA, USA) were negative for fish, and afterwards, she had eaten fish without any symptoms. However, two weeks ago, she experienced wheals and swelling on her eyelids immediately after ingesting the fish sausages again. Her medical history comprised atopic dermatitis, bronchial asthma, and allergic rhinitis.

The patient had negative results for specific IgE for cod, mackerel, and the parasite Anisakis using ImmunoCAP. The skin prick test (SPT) indicated a positive result for the eyeshadows 1% in saline (mean diameter of wheal, 4.4 mm) (Figure 1(b)) [6]. In SPTs with ingredients ingested in the two episodes of anaphylaxis, only fish sausage 10% in saline (7.4 mm) was positive. SPTs were positive for carmine (Wako, Richmond, VA, USA) 0.1% in phosphate buffered saline (PBS) (4.9 mm) and carminic acid (Wako) 0.1% in PBS (3.6 mm). She did not give her consent for challenge tests with fish sausages because she experienced severe anaphylaxis after ingesting the fish sausages. She was diagnosed with anaphylaxis due to cochineal contained in fish sausages and contact urticaria due to carmine contained in eyeshadows.

**Figure 2:**
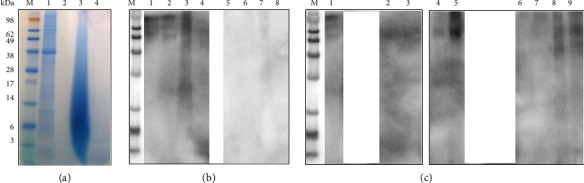
(a) SDS-PAGE of the fish sausage, the eyeshadow, the carmine, and the carminic acid. M, molecular mass standard; lane 1, eyeshadow extract; lane 2, fish sausage extract 1 mg/mL in phosphate buffered saline (PBS); lane 3, the carmine extract, 20 *μ*g/mL in PBS; and lane 4, the carminic acid, 20 *μ*g/mL in PBS. (b) IgE immunoblotting using the fish sausage, the eyeshadow, the carmine, and the carminic acid. M, molecular mass standard; lanes 1 and 5, the eyeshadow extract; lanes 2 and 6, the fish sausage extract; lanes 3 and 7, the carmine extract; and lanes 4 and 8, the carminic acid extract. We used the patient serum for lanes 1–4 and the control pooled serum from subjects without food allergy for lanes 5–8. The patient's IgE antibody was bound to the eyeshadow protein bands at the molecular weight of approximately 86, 114, and 130 kDa, which were the same as that of the fish sausage protein bands. In addition, IgE immunoblotting using the carmine showed the broad band at 86–130 kDa, whereas IgE immunoblotting using the carminic acid showed no band. These results indicated that the eyeshadow and the fish sausage could contain the same causative allergen as the carmine. On the other hand, IgE immunoblotting with the control pool serum shows no bands. (c) Evaluation of the allergenicity of the eyeshadow and the carmine after the chelation with EDTA. M, molecular mass standard; lanes 1–3, the eyeshadow extract; lanes 4, 6, and 7, the carmine extract, 20 *μ*g/mL; lanes 5, 8, and 9, the carmine extract, 40 *μ*g/mL. The eyeshadow and carmine extracts were applied to lanes 2, 6, and 8, respectively, after preincubation in the presence of the chelating agent, 5 mM ethylenediaminetetraacetic acid (EDTA). In addition, the eyeshadow and the carmine extracts were applied to lanes 3, 7, and 9, respectively, after preincubation in the presence of the chelating agent, 25 mM EDTA. The three protein bands at the molecular weight ranging 86–130 in the eyeshadow and the carmine extracts were diminished through preincubation in the presence of EDTA in a concentration-dependent manner. These results suggest that the causative allergen in the eyeshadow and the carmine lost its allergenicity through chelation with EDTA.

To identify causative allergens of fish sausages and eyeshadows, we performed IgE immunoblotting using fish sausages, eyeshadows, and carmine. In IgE-immunoblotting using the eyeshadow and fish sausage, the patient serum IgE was bound to three protein bands at approximately 86, 114, and 130 kDa (Figures 2(a) and 2(b)). In addition, IgE immunoblotting using the carmine showed a broad band at 86–130 kDa, which were consistent with those using the eyeshadow and fish sausage, whereas there is no band using CA. These results led the hypothesis that although CA does not have allergenicity, in production of carmine from CA by chelating, carmine would have allergenicity. To investigate the influence of chelation on carmine allergy, we performed IgE immunoblotting using ethylenediaminetetraacetic acid (EDTA). Interestingly, the protein bands using the eyeshadow and carmine were diminished by preincubation in the presence of EDTA as a chelating agent. The results indicated that the causative allergens of the carmine contained in eyeshadow could be dechelated by EDTA, reducing its allergenicity (Figure 2(c)).

## 3. Discussion

Carmine allergy is female-dominant, and the allergic symptoms were frequently associated with cosmetics use [7, 8]. In this case, the eyelid symptoms of contact urticaria caused by eyeshadows were similar to those of anaphylaxis from fish sausages. Therefore, we suspected that the patient was epicutaneously sensitised to carmine in the eyeshadows and afterwards experienced orally induced anaphylaxis after ingesting cochineal contained in the sausage.

In this case, in IgE immunoblotting, IgE-binding bands to protein at 86, 114, and 130 kDa, were common in the fish sausages, the eyeshadow, and the carmine. The three bands could be common causative allergens in the fish sausages, the eyeshadows, and the carmine. In addition, the three bands were diminished due to dechelation with EDTA. Although CA is a small molecule with a molecular weight of 492 Dalton, in the carmine production, chelation could confer allergenicity to CA by forming larger molecules containing epitopes. Our study is the first to demonstrate the effect of chelation on the allergenicity of carmine. However, further studies are needed in order to confirm whether this finding is common in carmine allergies.

In conclusion, carmine contained in cosmetics can cause epicutaneous sensitisation and consequently can induce food anaphylaxis. To prevent sensitisation in carmine allergy, the effect of chelation on allergenicity of carmine should be considered.

## Data Availability

No data were used to support this study.
